# Radiomics‐Driven Classification of Small White Matter Hyperintensities and Perivascular Spaces on T1‐Weighted MRI

**DOI:** 10.1002/alz70856_105126

**Published:** 2026-01-07

**Authors:** Maryam Fotouhi, Fardin Samadi Khoshe Mehr, Bino Varghese, Nasim Sheikh‐Bahaei, Jeiran Choupan

**Affiliations:** ^1^ Laboratory of Neuroimaging, USC Mark and Mary Stevens Neuroimaging and Informatics Institute, Keck School of Medicine, University of Southern California, Los Angeles, CA, USA; ^2^ Laboratory of Neuroimaging, USC Mark and Mary Stevens Neuroimaging and Informatics Institute, Keck School of Medicine, University of Southern California, Los Angles, CA, USA; ^3^ Keck School of Medicine, University of Southern California, Los Angeles, CA, USA

## Abstract

**Background:**

This study aims to develop a radiomics‐based classifier that integrates features related to shape, texture and intensity uniformity from T1W‐MRI scans to accurately differentiate between perivascular spaces (PVS) and small white matter hyperintensity (WMH) lesions on T1‐MRI.

**Method:**

A cohort of 1270 WMH and 2976 PVS lesions was extracted from the ADNI‐3 dataset and segmented using our previously validated workflow. We excluded PVS and WMH lesions with a voxel count beyond 500 to refine our model for the more challenging lesions. A total of 1223 radiomic characteristics were retrieved followed by feature selection approach, implementing a high‐correlation filter with a threshold of 0.8, yielding 282 remaining features. Subsequently, the LASSO technique was used to ascertain the ten most significant features. We used an ordinary least squares regression model to evaluate the importance of the chosen characteristics. Lesions were split across 80% training and 20% testing datasets. The classifier's optimum parameters were determined by 5‐fold cross‐validation, and the selected models were then assessed on the test dataset.

**Result:**

The first six features belong to first‐order frequency decompositions of the wavelet and showed significant differences within PVSs and WMHs (*p*‐value < 0.0001), suggesting that WMH lesions are denser than PVS lesions. The two shape‐related features, including elongation and sphericity, suggest significant differences in the growth pattern across each class (*p*‐value < 0.0001). The two selected features from GLRLM and GLDM indicate that PVS lesions have a more homogenous tissue structure compared to WMH lesions (*p*‐value<0.0001). The RF classifier outperformed other models, showed potency in the test dataset, achieving an accuracy of 0.95, sensitivity of 0.96, and specificity of 0.90.

**Conclusion:**

Differentiation of PVS and WMH lesions on T1W images can be challenging, particularly in the absence of T2‐FLAIR, due to their similar hypointense appearance. Our study aimed to bridge this gap, revealing that beyond the well‐established shape markers like elongation and sphericity, the homogeneity of intensity and variations in tissue texture also emerge as critical features in distinguishing these lesions. These findings suggest that a training classifier with the specific radiomics features from T1‐MRI could achieve high accuracy in classifying PVS and WMH lesions.

